# Categories and Profiles of Uveitis in Vogt-Koyanagi-Harada Syndrome With Systemic Correlation: Inferences From a Tertiary Multispecialty Hospital

**DOI:** 10.7759/cureus.64998

**Published:** 2024-07-20

**Authors:** Simran G Raichandani, Gowtham Kim, Radha Annamalai, Sudha Rangarajan, Rajeswari Sankaralingam

**Affiliations:** 1 Ophthalmology, Sri Ramachandra Institute of Higher Education and Research, Chennai, IND; 2 Ophthalmology, Aravind Eye Hospital, Chennai, IND; 3 Dermatology, Sri Ramachandra Institute of Higher Education and Research, Chennai, IND; 4 Rheumatology, Sri Ramachandra Institute of Higher Education and Research, Chennai, IND

**Keywords:** rheumatology and autoimmune diseases, dermatological manifestation, systemic steroids, multifocal choroiditis, fundus fluorescein angiography (ffa), posterior uveitis, serous retinal detachment, granulomatous panuveitis, vkh disease

## Abstract

Introduction

Vogt-Koyanagi-Harada (VKH) syndrome is a granulomatous, autoimmune panuveitis, affecting the eyes, ears, skin, and meninges. It can cause choroiditis and can progress to the retina and optic disc causing visual loss. Imaging using fundus fluorescein angiography (FFA), indocyanine green angiography (ICGA), and enhanced depth imaging-ocular coherence tomography (EDI-OCT) is required for clinical evaluation and management. Steroids and immunosuppression are the treatment modalities used.

Aim

The aim of this study is to report the correlation and severity of uveitis in relation to systemic manifestations.

Method

A retrospective study including 100 patients with VKH syndrome was carried out. They were classified based on clinical manifestations and investigations such as FFA, ICGA, B-scan ultrasonography (USG), and ocular coherence tomography (OCT). Patients were characterized as complete, incomplete, and probable VKH syndrome. Laboratory investigations were performed, and statistical analysis was done.

Results

Probable VKH syndrome was found to be the most common form of presentation in our study population. Defective vision was the most common complaint among the patients. Extraocular manifestations included tinnitus, vertigo, alopecia, headache, fatigue, and vitiligo and were seen in 33% of the patients. Disc edema and serous retinal detachment were seen in 85% of the patients. Improvement was noted in 25% of the patients with the use of corticosteroids.

Conclusion

Response to treatment with systemic corticosteroids and immunosuppression in the acute phase of uveitis is better compared to chronic uveitis. The ophthalmologist is usually first consulted in VKH syndrome due to presenting ocular complaints. A multidisciplinary approach is key to providing holistic management.

## Introduction

Vogt-Koyanagi-Harada (VKH) syndrome is a granulomatous, autoimmune panuveitis with the eyes, ears, skin, and meninges being affected and melanocytes being the target of inflammation [1]. In the eye, the choroidal stroma, which contains melanocytes, results in primary stromal choroiditis [2]. The inflammation can progress to affect the retina and the optic disc, thus causing severe visual loss, or may become chronic, which shows poor response to treatment [2]. It is believed to be common in the East Asian population, less common in Indians, and infrequent in Caucasians [2]. It was first reported in 1906 by Alfred Vogt in several patients in Japan. In 1914, Yoshizou published an article describing two patients with all the characteristic features of what is now known as VKH syndrome. In 1923, Einosuke Harada first described acute diffuse stromal choroiditis, which is the Harada component of VKH syndrome. A T-cell-mediated autoimmune response against melanocytes is considered as the etiopathogenesis of the condition [3-5]. In patients with VKH syndrome, mononuclear cells in the blood recognize tyrosinase-related peptides, which mediate melanin synthesis [3-5]. An immune reaction and inflammation are triggered when the antigen-derived peptides bound on human leukocyte antigen (HLA) molecules are recognized [3-5].

Conventional classification divided VKH syndrome into probable (only ocular disease), incomplete (ocular and integumentary involvement), and complete (ocular, integumentary, and neurological involvement) categories [1]. The requirement of diagnostic criteria based on disease resolution and prognosis has led to the development of two clinically relevant phenotypes, namely, acute or initial-onset VKH syndrome and chronic or recurrent VKH syndrome.

Clinically, inflammation starts in the choroidal stroma, but manifestation can be varied among different patients [2]. The subtypes, acute and chronic VKH syndrome, showed different responses to treatment and prognosis. Imaging characteristics using a combination of fundus fluorescein angiography (FFA), indocyanine green angiography (ICGA), and enhanced depth imaging-ocular coherence tomography (EDI-OCT) are required for clinical evaluation and successful management [2]. Corticosteroids and immunosuppression are required, but the utility in initial-onset and chronic stages must be reviewed for each patient [6].

In the prodromal stage, the patient can have integumentary, auditory, and neurological features, which include meningism, tinnitus, vertigo, sensorineural hearing loss, and hyperesthesia of the scalp [6,7]. At this stage, choroidal inflammation can be detected with the use of ICGA and EDI-OCT, and the evaluation of the cerebrospinal fluid (CSF) sample at this stage can aid in confirming the diagnosis. In the acute stage, the patient develops bilateral, asymmetrical panuveitis with serous retinal detachment and papillitis and when not treated can progress to involve the vitreous, ciliary body, and aqueous humor [7-9]. Treatment with high-dose steroids and immunosuppression must be started within 2-4 weeks of onset to prevent complications and chronicity [10]. Chronic VKH syndrome is characterized by sunset glow fundus, subretinal fibrosis, recurrent granulomatous anterior uveitis, the depigmentation of the retinal pigment epithelial cells, and Dalen-Fuchs nodules, which are granulomas formed by the aggregation of retinal pigment epithelium (RPE) and inflammatory cells [6,7]. Poor vision is associated with the development of complications such as cataract, secondary glaucoma, choroidal neovascular membranes (CNVM), subretinal fibrosis, and chorioretinal atrophy [7]. A wide spectrum of clinical presentation exists, and this study aims to determine the types of manifestations in VKH syndrome, the pattern of uveitis, visual loss, complications, and management in this population. This study also aims to correlate the systemic features with the incidence of ocular involvement in patients with VKH syndrome.

## Materials and methods

Study settings, study population, and inclusion and exclusion criteria

This study is a hospital-based, retrospective study, conducted at a multispecialty tertiary referral center in South India in liaison with the department of dermatology, which included 100 patients with an established diagnosis of VKH syndrome, which was the inclusion criteria of the study. Patients with a history of ocular trauma or surgery, any clinical or laboratory evidence of other ocular diseases, and a history of other systemic disorders causing uveitis such as connective tissue disorders were excluded from the study.

Ethical consideration

The study had been approved by the Institutional Research Ethics Committee of Sri Ramachandra Institute of Higher Education and Research, with number CSP-MED/24/MAR/100/77 (dated 28.05.2024).

Study period, study design, and study method

We analyzed data from the hospital records of all patients with VKH syndrome who presented to the ophthalmology department at our institute. Medical records of all patients were reviewed for demographic information, medical history, clinical characteristics, and laboratory findings. Among a total of 1000 uveitis patients seen in our institute over 10 years, from January 2013 to January 2023, VKH syndrome constituted 1% of them. The classification was based predominantly on clinical manifestations and supported by ancillary investigations such as fundus fluorescein angiography, indocyanine green angiography, B-scan ultrasonography (USG), and optical coherence tomography. The revised criteria given by the International Committee on Nomenclature were used to categorize patients as complete, incomplete, and probable VKH syndrome. Based on the Standardization of Uveitis Nomenclature Working Group classification criteria, a distinction between early and late stages was made. Ancillary ophthalmic investigations such as fundus photography, fundus fluorescein angiography, and B-scan ultrasonography that were performed were studied.

We used FFA (FF450 Plus, Carl Zeiss, Bengaluru, India), which was performed to identify early hypofluorescence and late hyperfluorescence in active lesions. OCT (Cirrus HD400, Carl Zeiss, Bengaluru, India) was performed in the initial stages to determine the level of lesion and complications and monitor progression and response to treatment. B-scan (Marvel B-scan, Appasamy Associates, Chennai, India) was used to identify choroidal thickness, vitreous activity, and the presence of retinal detachment.

Laboratory investigations such as complete blood counts, ESR, venereal disease research laboratory (VDRL), chest imaging, and polymerase chain reaction (PCR) were performed where there was a diagnostic dilemma.

All the data were collected and studied. Statistical analysis was performed using Pearson's chi-square test for categorical variables. Statistical analysis was performed with the Stata version 8.0 (StataCorp LLC, College Station, TX). A P value of 0.05 was considered statistically significant.

## Results

**Figure 1:**
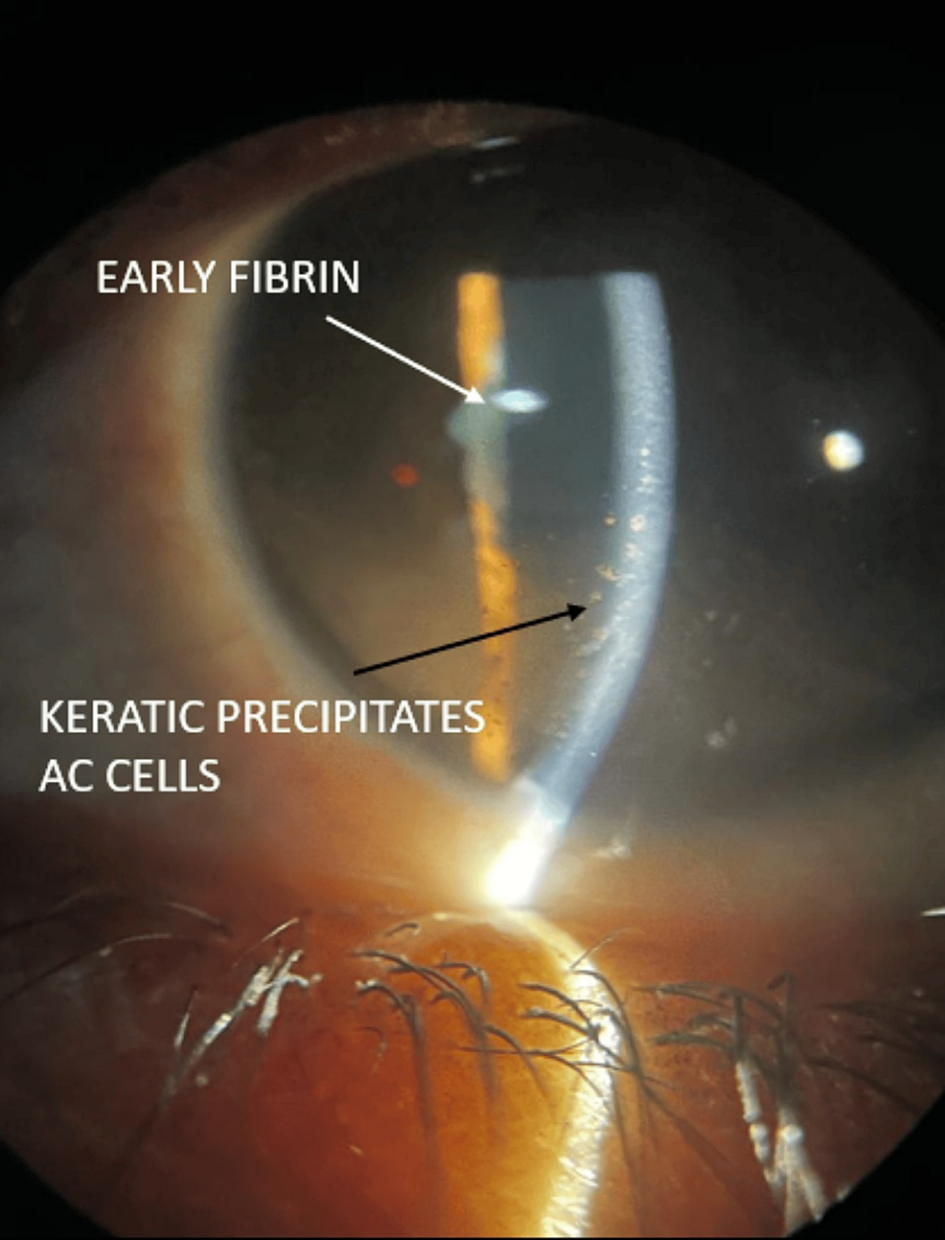
Granulomatous anterior uveitis. Granulomatous anterior uveitis characterized by anterior chamber (AC) cells, large mutton fat type of keratic precipitates on the corneal endothelium (black arrow), and fibrin membrane over the pupillary area (white arrow).

**Figure 2:**
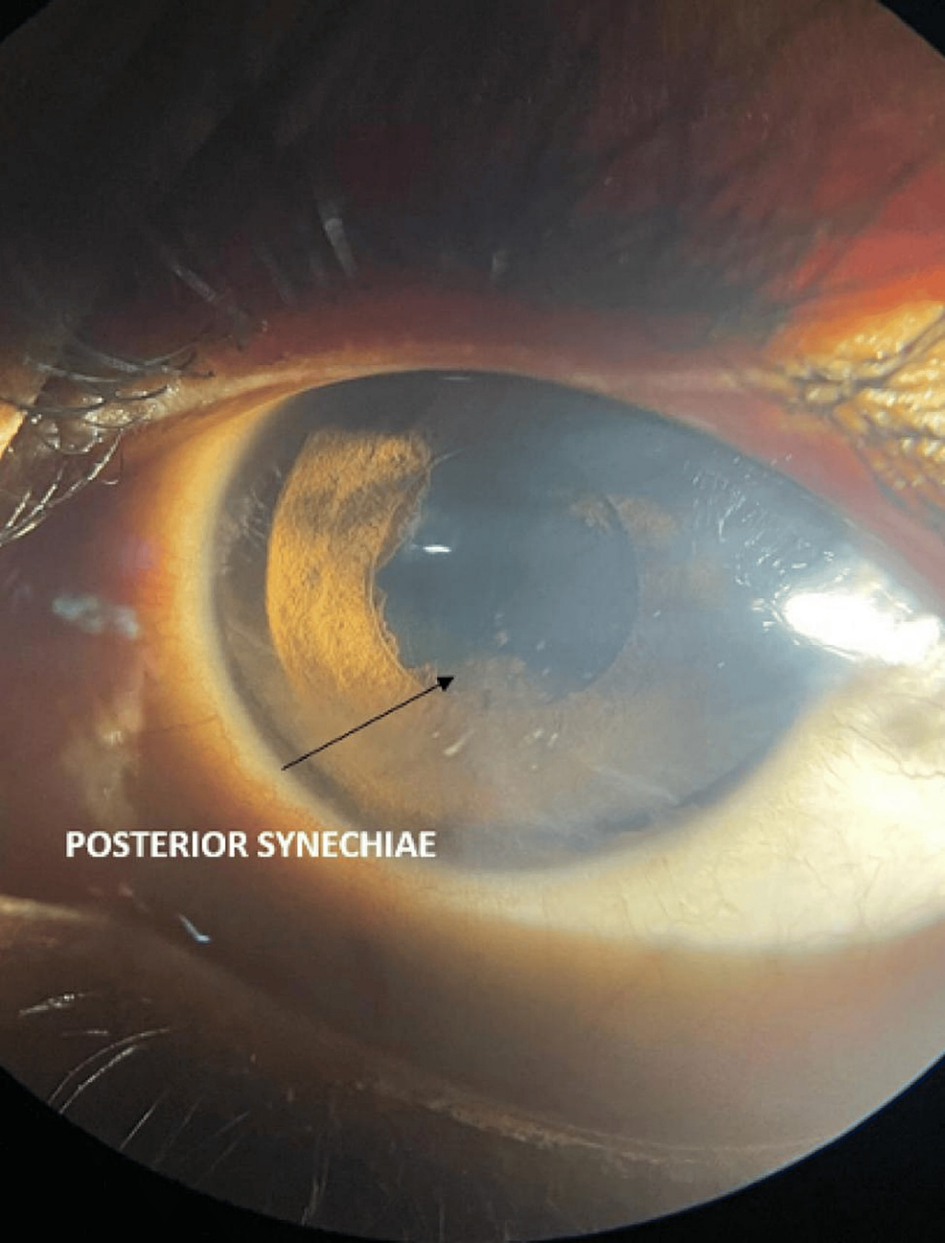
Posterior synechiae. Posterior synechiae in the inferior quadrant with mutton fat keratic precipitates suggestive of severe ocular inflammation.

Among our study cohort of 100 patients, 79% were females, and 21% were males. The mean age of our cohort was 45 years with a standard deviation of five years. Ocular complaints preceded systemic complaints in 82% of our patients. Defective vision was the most common complaint with severe reduction up to hand movements in 63% of the patients. The disease was bilateral and asymmetrical in 70% of the patients. Anterior segment examination revealed granulomatous anterior uveitis in 43 patients (52.4%) (Figures 1, 2).

**Figure 3:**
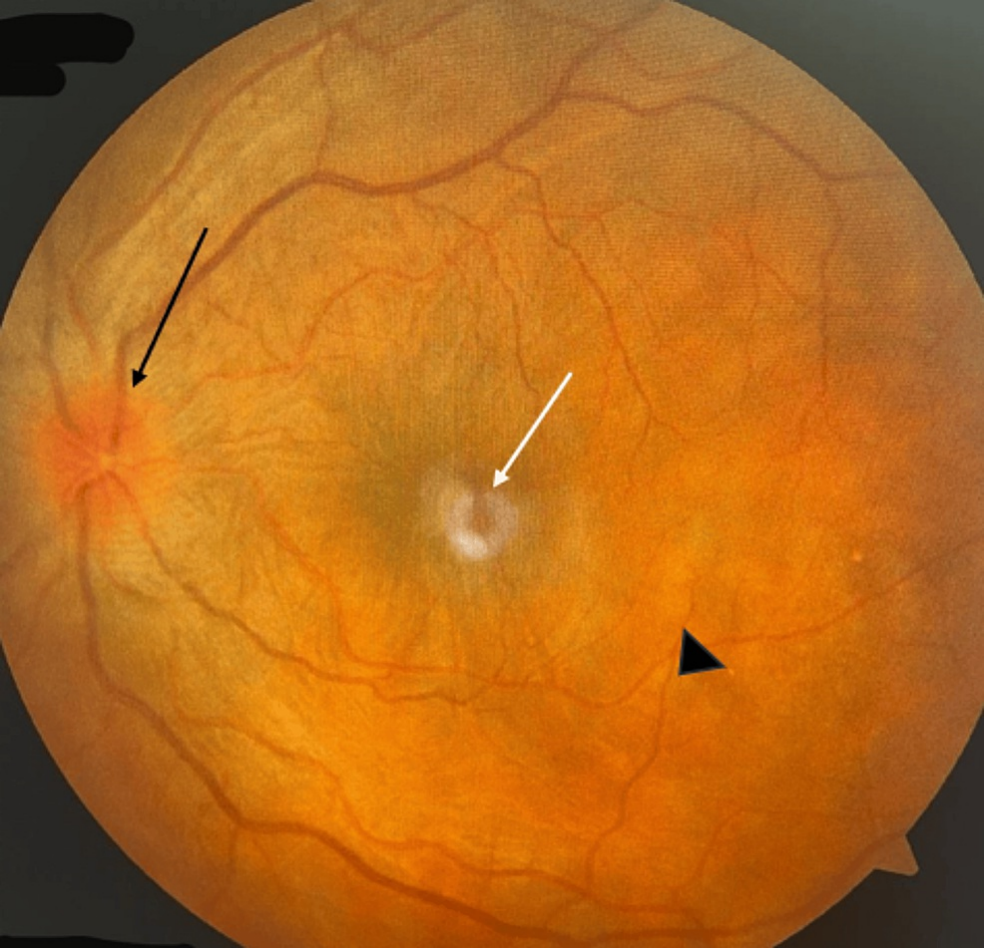
Fundus photo of the left eye. Fundus photo of the left eye showing hyperemic disc with a blurring of disc margins (black arrow), pigmentary changes at the macula (white arrow), and multifocal choroiditis patches with serous detachment (black arrowhead) suggestive of Vogt-Koyanagi-Harada syndrome.

The fundus examination revealed hyperemic disc edema in 85%, exudative retinal detachment in 22%, and diffuse pigmentary disturbance and retinal vasculitis in 8% of the patients (Table 1 and Figure 3).

**Table 1:** Fundus findings in our patients.

Fundus findings	N=100	Percentage
Hyperemic disc edema	85	85%
Exudative retinal detachment	22	22%
Serous retinal detachment	87	87%
Retinal vasculitis	8	8%
Diffuse pigmentary disturbance	8	8%

**Figure 4:**
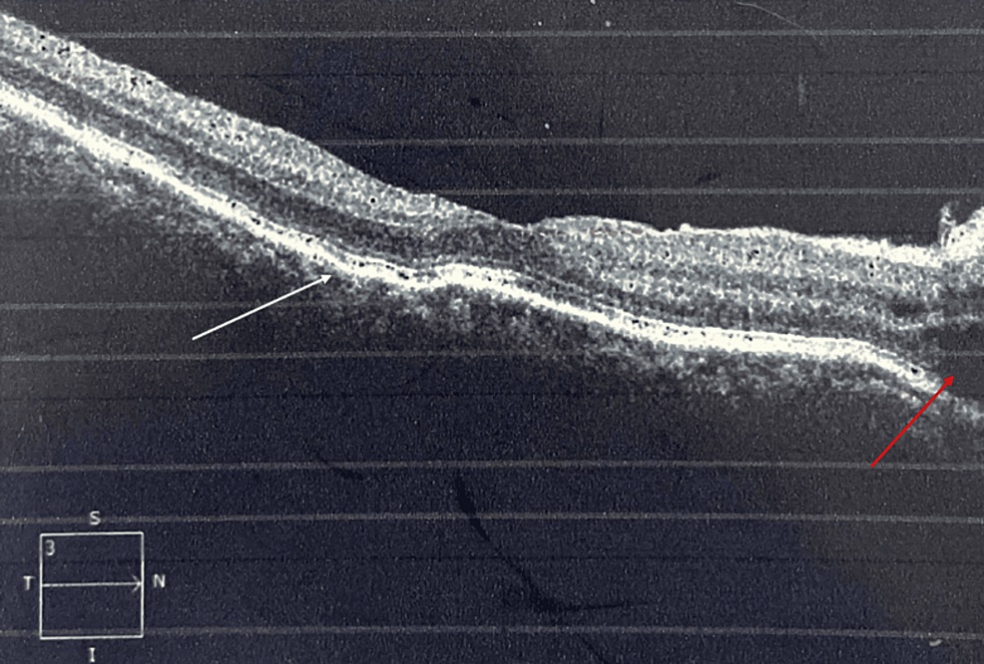
Ocular coherence tomography of the macula. Ocular coherence tomography of the macula region shows retinal pigment epithelial folds (white arrow) and a serous retinal detachment (red arrow), which are characteristics of Vogt-Koyanagi-Harada syndrome.

**Figure 5:**
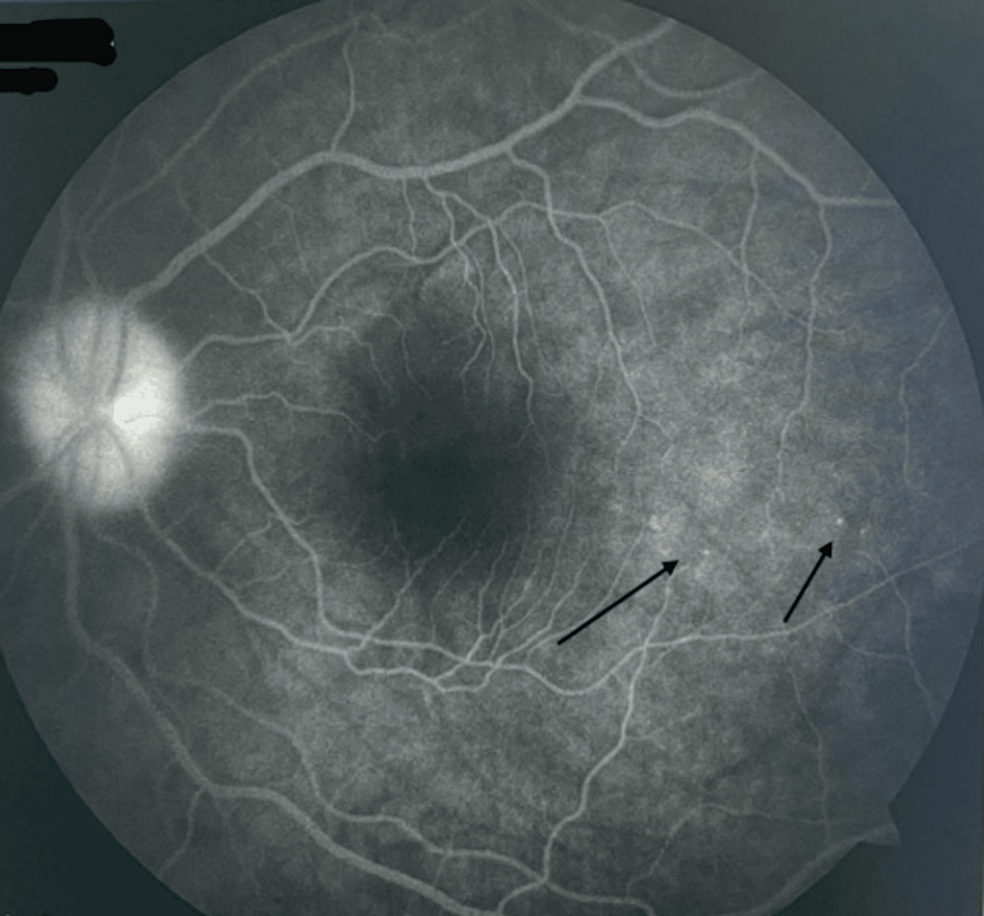
Fundus fluorescein angiography showing pinpoint leaks (black arrows).

Extraocular manifestations occurred only in one-third (33%) of the patients. Tinnitus and vertigo were the most frequent extraocular manifestation and were seen in 16 patients (48%), followed by alopecia in nine patients (27.3%), headache and fatigue in six patients (18.2%), and vitiligo in two patients (6%) (Table 2). Among our study cohort, 18% of the patients had come with a recurrent episode, and 72% of the patients had the first episode. B-scan ultrasound was performed in all patients, and choroidal thickness was found to be above 325±23 microns. OCT showed multifocal serous retinal detachment in 87% of the patients and hyperreflectivity in 71% of the patients (Figure 4). FFA showed pinpoint hyperfluorescence in the early phase with increasing intensity and leakage and staining in the late phases (Figure 5). Vascular leakage was noted in 6% of the patients. Corticosteroids were given in the form of oral prednisolone 1 mg/kg body weight in 63 patients, and combined corticosteroids and immunosuppressives were started in 37 patients. In 27 patients, 1 m per day of intravenous methylprednisolone was given in an ICU setup. The immunosuppressives used were azathioprine 50 mg three times daily or methotrexate 8 mg/week. Injection adalimumab 80 mg subcutaneously, followed by 40 mg at a two-week follow-up, was used to control VKH syndrome in seven patients. Following immunosuppression, 76 patients improved, and 14 patients deteriorated. Vitrectomy in three patients and cataract surgery in 81 patients were performed. The earliest improvement was noted at a one-month follow-up. All patients had a minimum follow-up of one year, and during this time, vision was maintained between 6/24 and 6/9 in 89 patients. Complications developed in 67 patients, of whom cataracts developed in 46 patients (68%), epiretinal membrane in five patients (7.4%), and subretinal fibrosis with retinal atrophy in three patients (4.5%) (Table 3).

**Table 2:** Extraocular manifestations of Vogt-Koyanagi-Harada syndrome.

Extraocular symptoms	N=33	Percentage
Tinnitus and vertigo	16	48%
Alopecia	9	27.3%
Headache and fatigue	6	18.2%
Vitiligo	2	6%

**Table 3:** Vision-threatening complications.

Complications	N=67	Percentage
Complicated cataract	46	68%
Epiretinal membrane	5	7.4%
Subretinal fibrosis with retinal atrophy	3	4.5%

## Discussion

This is a retrospective study on a cohort of patients with VKH syndrome conducted in a multispecialty tertiary referral center. This is one of the first large single-center studies to report a large series of VKH syndrome cases in India and the correlation and severity of uveitis with systemic manifestations. This study has addressed the clinical, imaging, and histopathological perspectives with systemic features of VKH syndrome. It has been described in Latinx and East Asian populations. The incidence of VKH syndrome was 10% among all patients presenting with uveitis and was more common in females with a mean age of 45 years.

Probable VKH syndrome is the most common presentation based on our study population. Complete VKH syndrome was rare, and because of the absence of a complete presentation, fewer patients were diagnosed in the early stages. Extraocular manifestations were seen in 33% of the patients; among these, auditory features such as tinnitus were more common than neurological and integumentary features. This was not in concurrence with previous reports that state headache and photophobia as the most common extraocular manifestation [9-13]. As described by Lavezzo et al. [10] and Ohno et al. [11], the meningeal involvement was variable and presented with features such as clinical meningism. Dermatological features such as alopecia, vitiligo, and poliosis were found to be associated with chronic disease [9,10], and these findings were similar to findings noted by Rao et al. [13].

Patients' response to treatment with a combined regimen comprising immunosuppressants and steroids that were started early was found to be better. In the four studies on combined regimens (steroids with immunosuppressants) in our literature review, several different immunosuppressants were administered, and all had similarly good results [14-17]. This suggests that the choice of immunosuppressants does not matter as much as the choice of giving corticosteroids with immunosuppressant combined therapy. Macular involvement due to serous retinal detachment was the most common cause of severe visual loss. Patients with macular involvement due to serous retinal detachment were initially treated with intravenous methylprednisolone 1 g/day for three days in an ICU setup, followed by oral corticosteroids along with immunosuppressants such as azathioprine 50 mg three times a day.

Treatment must be started within 3-4 weeks to prevent progression to chronic disease. This stage is characterized by the depigmentation and development of the characteristic sunset glow fundus or more severe subretinal fibrosis [7,9,10,18].

Limitations

This study being a retrospective study relies on the accuracy and completeness of information documented in medical records. So, there is a possibility of underreporting or missing data. This study's single-center design may also limit the generalizability of the findings to other regions or healthcare institutions with a different population of patients. Serial imaging was not done for the patients to monitor their response to therapy. This study does not compare the efficacy of different immunosuppressants used among VKH syndrome patients.

## Conclusions

The primary aim of this study was to determine the types of manifestations in VKH syndrome, the pattern of uveitis, visual loss, complications, and management in this population and to correlate it with systemic manifestations of the disease. This study was undertaken to identify the risk of visual loss in VKH syndrome patients who are being treated for the dermatological and auditory components of the disease. VKH syndrome is a relatively rare disorder, and ours is one of the first papers to describe in detail the clinical, imaging, and histopathological perspectives with systemic features in a large series of VKH syndrome cases in India. In our study population, probable VKH syndrome was the most common presentation, and serous macular detachment was the most common cause of visual loss. Response to treatment with systemic corticosteroids and immunosuppression in the acute phase of uveitis is better compared to recurrent or chronic uveitis due to complications including complicated cataract, secondary glaucoma, subretinal fibrosis, and retinal atrophy. The ophthalmologist is usually first consulted in VKH syndrome due to ocular complaints. A multidisciplinary approach is key to providing holistic management.
